# Investigation of Biological Factors Contributing to Individual Variation in Viral Titer after Oral Infection of *Aedes aegypti* Mosquitoes by Sindbis Virus

**DOI:** 10.3390/v14010131

**Published:** 2022-01-12

**Authors:** Peter Hodoameda, Linus Addae, Rollie J. Clem

**Affiliations:** 1 Division of Biology, Kansas State University, Manhattan, KS 66506, USA; hodoameda@ksu.edu; 2 Department of Statistics, Kansas State University, Manhattan, KS 66506, USA; laddae@ksu.edu

**Keywords:** Sindbis virus, *Aedes aegypti*, biological variation, oral infection, vector competence, mosquito midgut

## Abstract

The mechanisms involved in determining arbovirus vector competence, or the ability of an arbovirus to infect and be transmitted by an arthropod vector, are still incompletely understood. It is well known that vector competence for a particular arbovirus can vary widely among different populations of a mosquito species, which is generally attributed to genetic differences between populations. What is less understood is the considerable variability (up to several logs) that is routinely observed in the virus titer between individual mosquitoes in a single experiment, even in mosquitoes from highly inbred lines. This extreme degree of variation in the virus titer between individual mosquitoes has been largely ignored in past studies. We investigated which biological factors can affect titer variation between individual mosquitoes of a laboratory strain of *Aedes aegypti*, the Orlando strain, after Sindbis virus infection. Greater titer variation was observed after oral versus intrathoracic infection, suggesting that the midgut barrier contributes to titer variability. Among the other factors tested, only the length of the incubation period affected the degree of titer variability, while virus strain, mosquito strain, mosquito age, mosquito weight, amount of blood ingested, and virus concentration in the blood meal had no discernible effect. We also observed differences in culture adaptability and in the ability to orally infect mosquitoes between virus populations obtained from low and high titer mosquitoes, suggesting that founder effects may affect the virus titer in individual mosquitoes, although other explanations also remain possible.

## 1. Introduction

Arboviral diseases account for approximately 17% of all infectious diseases globally and approximately 40,000 deaths each year, making them a great public health concern [1]. Arboviruses of medical importance include chikungunya virus, dengue virus (DENV), Zika virus, West Nile virus, yellow fever virus, Sindbis virus (SINV), and many others [1].

SINV is classified as an emerging pathogen that is mainly reported in northern Europe and southern Africa [2]. In Sweden, disease caused by SINV infection in humans is called Ockelbo disease, which is characterized by rash, arthritis, and mild fever [3]. Ockelbo disease is not fatal, and most patients recover within weeks or months, but arthralgia and myalgia can persist for years following infection, which suggests an inflammatory response or a persistent infection [3]. The prevalence of SINV infection is mostly low, resulting in short and sporadic epidemics [4].

Arboviral diseases continue to persist due to the existence of the vector mosquitoes in endemic areas [5] and also due to the ability of these mosquitoes to spread to new environments to transmit the viruses [6,7]. A major factor contributing to the persistence of mosquitoes is a lack of robust vector control strategies [8]. The inefficiency of current vector control strategies is due to a combination of factors including insufficient resources, a lack of technical know-how [9], and the development of mosquito resistance to chemical insecticides [10], especially in developing countries where the disease burden is highest [9]. Also, the absence of effective antiviral drugs for the treatment of arboviral diseases and vaccines against most arboviruses contributes to the high morbidity and mortality caused by these viruses.

A major limiting factor hindering the effort to develop new vector control strategies is the lack of a comprehensive understanding of what determines vector competence. Many studies have identified biological factors that contribute to differences in mosquito susceptibility to arbovirus infection, such as mosquito age [11], virus strain [12], mosquito population or strain, virus concentration in host blood [13], mosquito size [14], and incubation period (the amount of time after exposure to the virus-containing blood meal) [15]. However, an area of study that has been largely ignored is the extreme variation in the levels of viral replication that is observed amongst individual mosquitoes in a single experiment. We and many others have documented that a high degree of variation in viral titer exists between individual mosquitoes following oral infection, even between individual mosquitoes of inbred mosquito strains that have been raised in the same environmental conditions, and subsequently orally fed with the same virally infected blood [16,17,18,19]. A large variation in the virus titer between individual mosquitoes has been documented numerous times, but it is usually ignored by researchers, and the causes of this variation are largely unaddressed. One study examined individual *Ae. aegypti* from field-caught populations after infection with DENV and correlated differentially expressed genes with the levels of viral RNA [20], resulting in the identification of 39 candidate genes that were proposed to affect DENV replication. However, since these mosquitoes were from a wild population, they would be expected to have a relatively large amount of genetic diversity, which likely contributed to the variation in titer.

In this study, we used SINV as a model arbovirus to investigate the contributions of several biological factors to variation in virus titer using the Orlando strain of *Ae. aegypti*, a laboratory strain that was first established around 1940 and has been highly inbred since that time. Although precise husbandry records are not available, it has been reported that occasional outcrossing of Orlando with wild *Ae. aegypti* populations was performed up to 1992 [21]. We were able to confirm that the mosquito line used in this study has not been outcrossed since at least 2003 and likely for longer (Alden Estep, USDA-ARS, personal communication). The biological factors we investigated included mosquito strain, virus strain, mosquito age and size, virus concentration in the blood meal, the amount of blood ingested, and incubation period. To the best of our knowledge, this is the first study investigating which biological factors contribute to variation in virus titer between individual mosquitoes in a highly inbred mosquito line, where genetic variation between individuals is expected to be minimal.

## 2. Materials and Methods

### 2.1. Cell Lines

BHK-21 cells were propagated in Dulbecco modified Eagle medium (DMEM, Gibco, Waltham MA) supplemented with 10% fetal bovine serum (FBS, Atlanta Biologicals, Minneapolis, MN) at 37 °C with 5% CO_2_. C6/36 cells were maintained in Leibovitz’s L-15 media (Gibco) plus 10% FBS at 27 °C. Aag2 cells were maintained in Schneider’s media (Gibco) plus 10% FBS at 27 °C.

### 2.2. Mosquito Rearing

*Ae. aegypti* mosquito strains Orlando (obtained in 2005 from James Becnel, Agricultural Research Service, U.S. Department of Agriculture, Gainesville, FL, USA) and Rex-D (obtained in 2018 from Alexander Franz, University of Missouri) were reared at 27 °C, 80% humidity on a 12 h light/12 h dark cycle. *Ae. aegypti* eggs were hatched in water containing brain heart infusion media (BHI) for 2 days to produce mosquito larvae. Mosquito larvae were separated into pans (each pan containing approximately 100 larvae) with a small amount of BHI and ground fish meal added. Larvae metamorphosized into pupae within 5–7 days. The pupae were transferred into pupa cups and transferred into mosquito cages. Adult mosquitoes emerged beginning at around 2 days after transfer. Adults were allowed to feed on raisins and water before and after blood feeding. All mosquito experiments were performed in an arthropod containment level 2 insectary at Kansas State University.

### 2.3. MRE-16 and TE RNA Generation and Cell Transfection

The MRE-16 and TE infectious clones p5′dsMRE16ic and pTE5′2J, respectively, were obtained from Ken Olson (Colorado State University), and their construction was described [22,23]. The p5′dsMRE16ic plasmid was linearized with Ascl, while the pTE5′2J plasmid was linearized with Xhol. Capped viral RNA was produced from the linearized plasmids using m7 G(5′)ppp(5′)G Cap Analog (Ambion, Austin TX) and MEGAscribe SP6 Transcription Kit (Thermo Fisher, Waltham MA). A density of 5 × 10^5^ cells BHK21 cells were plated in each well of 6-well plates in serum-free opti-MEM media (Gibco) and allowed to attach for 2 h. Six μL of Lipofectamine 3000 (Thermo Fisher), 100 μL opti-MEM, and 10 μL of 13.5 ng/ul viral RNA were mixed and allowed to sit for 5 min. After incubation, 900 μL of opti-MEM was then added to the tube containing the mixture and mixed with the seeded BHK-21 cells [24]. The BHK-21 cells were incubated at 37 °C for 3 days. At 3 days post-transfection, the medium was collected, aliquoted, and stored as P1 virus stock at −80 °C. The P1 virus stocks were passaged by adding 200 μL of P1 virus to a T75 flask containing 90% confluent C6/36 cells and cultured in Leibovitz’s L-15 medium supplemented with 10% (vol/vol) FBS to obtain P2 viral stocks. At 5 days post-infection (dpi), the P2 virus was harvested, aliquoted, and stored at −80 °C. Viral titers were determined using median tissue culture infectious dose (TCID_50_) assay in BHK-21 cells, as described below. Virus stocks used in this study were thawed only once before use.

### 2.4. TCID_50_ Assay

A total of 1 × 10^4^ BHK-21 cells were seeded in each well of 96-well tissue culture plates. The DMEM media was supplemented with 15 μg/mL of penicillin/streptomycin (Invitrogen, Waltham MA). Frozen mosquito samples were thawed on ice and centrifuged at 4 °C for 3 min at 15,000× *g* to remove debris, while samples obtained from tissue culture cells were thawed on ice without centrifugation. Serial dilutions of each sample were added to 5 duplicate wells of BHK-21 cells. The plates were scored for cytopathic effects after 5 days, and the proportion of infected wells was used to calculate TCID_50_/_mL_ values [25]. The TCID_50_/_mL_ values were multiplied by 0.69 to obtain the PFU/mL values.

### 2.5. Oral Infection with SINV

Newly emerged adult female mosquitoes were allowed to feed on raisins and water ad libitum. At 2 days post-emergence, raisins were removed but not water, and mosquitoes were starved for 1 day. For the mosquito age experiment, mosquitoes ages 3, 5, 7, 14, and 21 days post-emergence were used, and they were starved a day before they turned the required age needed for the experiment. The mosquitoes were then separated into smaller containers. P2 virus stocks were mixed with defibrinated sheep blood in a ratio of 1:1 and fed to the mosquitoes for 1 h using a Hemotek membrane feeder (Hemotek Ltd., Blackburn, UK) covered with Parafilm. For the viral concentration experiment, the P2 virus stock was diluted with Leibovitz’s medium to the desired concentration. The mosquitoes were briefly cold-shocked at 4 °C, and then fully engorged females were separated from the remaining mosquitoes. Fed females were subsequently maintained on raisins and water for 5 days post-blood meal (PBM) before dissecting. For the incubation period experiment, mosquitoes were harvested at 5, 10, and 15 days PBM. At the appropriate time points, mosquitoes were killed by freezing at −20 °C, and the killed mosquitoes were placed in 200 μL DMEM plus 10% FBS and homogenized in 1.5-mL microcentrifuge tubes using disposable plastic pestles. The samples were frozen at −80 °C until the titers were determined by TCID_50_ assay.

### 2.6. Intrathoracic Injection of SINV

Newly emerged adult female mosquitoes were fed on raisins and water until they were 3, 7, or 10 days old. These female mosquitoes were cold anesthetized and injected with one pulse of 69 nl of MRE-16 P2 virus stock and diluted with DMEM plus 10% FBS so that each mosquito received a dose of 10 PFU, using a Nanoject II injector (Drummond Scientific Company, Broomall, PA, USA). After recovering, the mosquitoes were maintained on raisins and water for 5 days, after which they were harvested and titered as described above.

### 2.7. Virus Growth Curves and Mosquito Reinfections

Virus that was obtained from pools of five mosquitoes that exhibited high titer (2.0 × 10^8^ PFU/mL), medium titer (2.9 × 10^6^ PFU/mL), or low titer (2.1 × 10^4^ PFU/mL), or MRE-16 P2 stock virus were used to infect C6/36 and Aag2 cells at a multiplicity of infection (MOI) of 0.1 in a 6 well plate. The diluent used for high and medium titer stocks was prepared by homogenizing two female mosquitoes in 900 µl of Leibovitz’s (for infecting C6/36 cells) or Schneider’s (for infecting Aag2 cells) medium. After a 1 h absorption period, 2 mL of Leibovitz’s or Schneider’s medium containing 10% FBS was added into each well. At 0, 1, 2, 3, 4, and 5 days post-infection, 200 μL of cell culture medium containing virus were collected and frozen at -80 °C until ready for TCID_50_ assay.

### 2.8. Statistical Analysis

GraphPad Prism 5 software was used for statistical analysis utilizing Mann–Whitney U, Kruskal–Wallis, Fisher’s exact, two-way ANOVA (analysis of variance), and Pearson’s chi-square tests. Dunn’s test was used for post hoc multiple-comparison analysis for Kruskal–Wallis tests that showed significance difference. A *p*-value of < 0.05 was considered significantly different. Since the data were not normally distributed, interquartile range (IQR) was used rather than standard deviation to measure variation within each set of data.

## 3. Results

### 3.1. Greater Variation in Virus Titer Is Observed after Oral Infection Than Intrathoracic Infection

**Figure 1:**
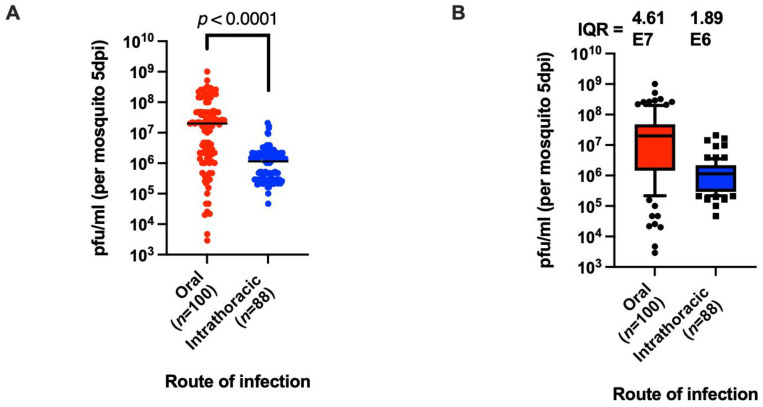
Oral versus intrathoracic infection of *Ae. aegypti.* (**A**) Titers of mosquitoes after oral or intrathoracic infection at 5 days post-virus exposure. (**B**) The same dataset as in (**A**) but displayed using box and whisker plots showing interquartile range (IQR) as a measure of variation. The whiskers represent the data range between 10% and 90%; the box represents the IQR, and the line inside the box is the median. The data points lying above and below the whiskers are defined as outliers. The sample size used in the oral infection was 100, while the sample size for intrathoracic infection was 88. Mann–Whitney test was used to analyze the results in (**A**).

The midgut is the first tissue to become infected when a mosquito takes a blood meal from an infected host and is thought to play a major role in determining vector competence due to the presence of both midgut infection and midgut escape barriers that must be overcome by the virus. However, mosquitoes can be artificially infected by injecting the virus directly into the hemocoel, thus bypassing these midgut barriers. To determine the effect of midgut infection on variation in the titer, Orlando mosquitoes were either allowed to feed on a blood meal containing 1.1 × 10^9^ PFU/mL or were injected with 10 PFU of passage 2 (P2) virus derived from an infectious clone of the MRE-16 strain of SINV, and then the virus titer in whole mosquitoes was determined by TCID_50_ assay at 5 days after virus exposure. The results of this experiment revealed a significantly higher median titer in mosquitoes at this time point after oral infection compared to intrathoracic infection (Fig. 1A, *p*-value < 0.0001). More interestingly, however, there was greater variation in the individual titers of orally infected mosquitoes than injected mosquitoes. The median titer of orally infected mosquitoes was 2.0 × 10^7^ PFU/mL with an interquartile range (IQR) of 4.61 × 10^7^, while the median titer for intrathoracic infection was 1.15 × 10^6^ PFU/mL with an IQR of 1.89 × 10^6^. The overall titer range for oral infection was around 10^3^–10^9^ PFU/mL (100,000-fold), while that for intrathoracic infection was around 10^5^–10^6^ PFU/mL (100-fold) (Figure 1A,B). Similar levels of variation following oral infection were observed in two additional replicates using mosquitoes from different egg batches (Figure S1). Together these results suggest that infection of the midgut and the resulting bottlenecks associated with midgut infection and midgut escape can have a strong effect on the resulting overall titer of the virus in individual mosquitoes, resulting in a high degree of variation in titer even in a highly inbred laboratory strain of mosquitoes.

### 3.2. Mosquito Age Affects the Median Titer but Not the Variability or Prevalence of Infection after Oral Infection

**Figure 2:**
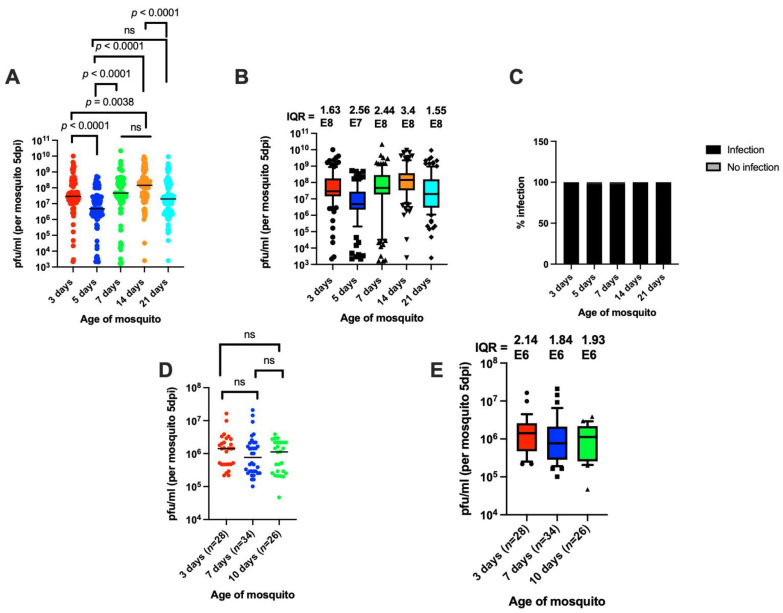
Oral and intrathoracic infection of *Ae. aegypti* adults of different ages (days post-emergence). (**A**) Titers of mosquitoes of different ages at 5 days PBM. (**B**) The use of IQR as a measure of variation. The dataset used in panel B is the same as in A. (**C**) Prevalence of infection of mosquitoes of different ages after oral infection. Sample size in (**A**–**C**) was 100 per treatment. (**D**) Titers of mosquitoes of different ages at 5 days after intrathoracic infection. (**E**) The use of IQR as a measure of variation. The dataset used in panel (**D**) is same as in (**E**). Sample size in (**D**,**E**) is shown below the graphs. Kruskal–Wallis test was used for statistical analysis in (**A**,**D**), while Dunn’s test was used for multiple comparison in (**A**). Chi-square test was used for analysis in (**C**). ns, non-significant.

One factor that is known to affect the mosquito immune response is the age of the mosquito [11,26]. To examine whether the age of mosquitoes has an effect on titer variability, mosquitoes of different ages (3, 5, 7, 14, and 21 days post-eclosion) were orally infected with MRE-16 at a titer of 1.1 × 10^9^ PFU/mL and then titered at 5 days post-blood meal (PBM). The results from this experiment show that mosquito age had a significant effect on median titer after oral infection (Figure 2A), although not in a consistent pattern. However, neither the amount of variation in titer nor the prevalence of infection was significantly affected by mosquito age (Figure 2B, C). This result shows that although the age of mosquitoes can affect the median titer, it does not affect the amount of variation in the viral titer in individual mosquitoes after oral infection.

We also examined whether the length of the mosquito developmental cycle had any effect on titer variation. We normally used female mosquitoes that emerged as adults between 2 and 7 days after the pupae were collected. In this experiment, we compared adults that emerged on days 2, 4, 5, 6, or 7 after pupae collection. These mosquitoes were allowed to feed on blood containing MRE-16 at a concentration of 1.1 × 10^9^ PFU/mL, and the virus titers were determined at 5 days PBM. Our results indicate that the length of the developmental cycle did not affect the median virus titer, infection prevalence, or titer variation (Figure S2).

### 3.3. Individual Titer Variation Is Also Independent of Mosquito Age after Intrathoracic Infection

To determine whether mosquito age affects titer variation when the virus is delivered intrathoracially, we injected mosquitoes of ages 3, 7, and 10 days post-eclosion with 10 PFU of MRE-16 and measured the virus titer at 5 days post-injection. All injected mosquitoes had detectable levels of virus, indicating no effect of age on infection prevalence. The results further indicate there were no significant differences in median titer or in the amount of titer variation between the different ages of mosquitoes after intrathoracic injection (Figure 2D,E). These results further support the conclusion that mosquito age does not affect variation in SINV titer between individual mosquitoes.

### 3.4. Mosquito Strain and Virus Strain Affect Median Titer but Not Individual Variation, While Virus Strain Also Influences Infection Prevalence

**Figure 3:**
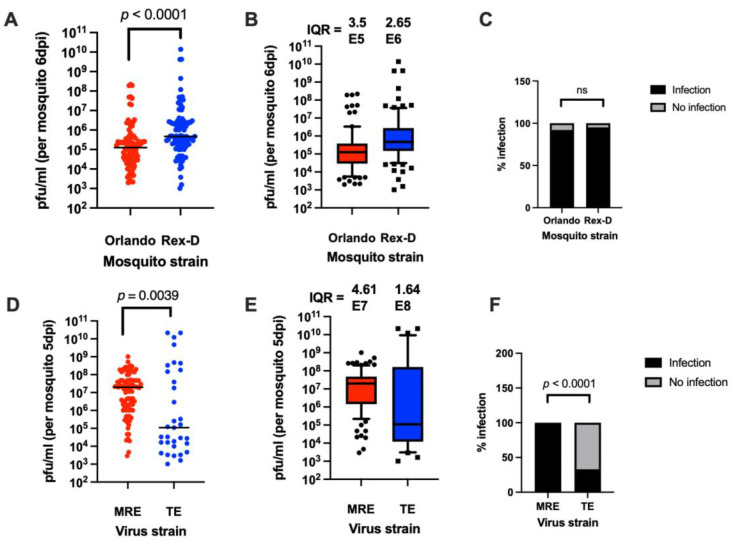
Oral infection of *Ae. aegypti* using either different mosquito strains or SINV strains. (**A**) Titers of Orlando and Rex-D mosquitoes at 5 days after feeding on a blood meal containing MRE-16. (**B**) The use of IQR as a measure of variation. The dataset used in panel B is the same as in (**A**). (**C**) Prevalence of infection of different mosquito strains after oral infection. (**D**) Titers of mosquitoes at 5 days PBM that were infected with MRE-16 or TE. (**E**) The use of IQR as a measure of variation. The dataset used in panel (**E**) is the same as (**D**). (**F**) Prevalence of infection after oral infection with different viral strains. Sample size was 100 for MRE and 33 for TE. Statistical analysis was performed as described for Figure 1. Fisher’s exact test was used for statistical analysis in (**C**,**F**).

Both mosquito strain and virus strain have been reported to affect vector competence [12,13]. To determine whether a large amount of variation in virus titer between individual mosquitoes is observed in more than one mosquito line, MRE-16 SINV (1.1 × 10^9^ PFU/mL) was used to orally infect mosquitoes from either the Orlando line or another laboratory *Ae. aegypti* line, Rex-D (Figure 3A–C). A significant difference in median virus titer was observed between the two mosquito lines. However, there was a large variation observed in both lines, and infection prevalence was also similar.

To test whether the virus strain can affect variation in titer, the MRE-16 strain was compared to another SINV strain, TE. The TE strain is more tissue culture-adapted than MRE-16 and is thus less efficient at causing oral infection in *Ae. aegypti* [22,23]. Orlando mosquitoes that had been fed 1.1 × 10^9^ PFU/mL MRE-16 or TE differed significantly in both median virus titer and infection prevalence (Figure 3D–F). However, both virus strains caused large variations in titer, with TE having an even higher IQR value than MRE-16. These results indicate that a large variation in SINV titer following oral infection occurs in more than one mosquito line or virus strain.

### 3.5. Both Virus Concentration and Incubation Period Affect Median Titer and Infection Prevalence, but Only Incubation Period Affects Titer Variation

**Figure 4:**
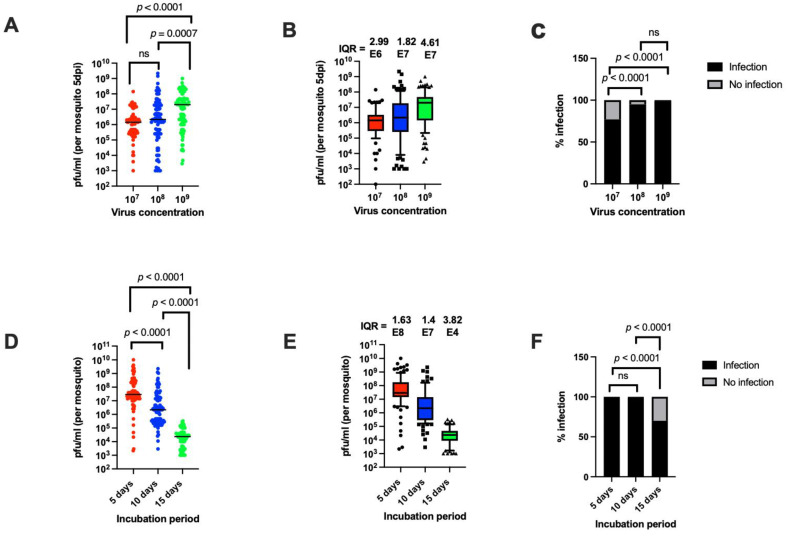
Oral infection of *Ae. aegypti* using different virus concentrations or incubation periods. (**A**) Titers of mosquitoes at 5 days PBM, after being allowed to feed on blood containing different virus concentrations. (**B**) The use of IQR as a measure of variation. The dataset used in panel (**B**) is the same as in (**A**). (**C**) Prevalence of infection of different mosquito strains after oral infection with different virus concentrations. Sample size was 100 per treatment. (**D**) Titers of orally infected mosquitoes after different incubation periods. (**E**) The use of IQR as a measure of variation. The dataset used in panel (**E**) is the same as in (**D**). (**F**) Prevalence of infection of mosquitoes with different incubation periods. Sample size was 100 per treatment. Statistical analysis was performed as described for Figure 2.

Virus concentration in the blood meal and incubation period (the amount of time after infection) have both been reported to affect the susceptibility of mosquitoes to arbovirus infection [12,13,15]. To assess whether the virus concentration or incubation period can affect titer variation, Orlando mosquitoes were allowed to feed on blood meals containing MRE-16 titers of 1.5 × 10^7^, 1.0 × 10^8^ or 1.1 × 10^9^ PFU/mL. Virus concentration significantly affected the median virus titer at 5 days PBM, with the median titer correlating with increased SINV concentration (Figure 4A,B). Infection prevalence was also significantly affected by viral concentration, with the prevalence being significantly lower at a concentration of 10^7^ PFU/mL than at the higher concentrations (Figure 4C). However, there was a large amount of variation in titer regardless of the virus concentration in the blood meal (Figure 4A,B).

To examine the effect of incubation period, Orlando mosquitoes were infected with MRE-16 at a titer of 1.1 × 10^9^ PFU/mL and incubated for 5, 10, or 15 days. Results from this experiment showed that the incubation period significantly affected the median viral titer, with the median titer dropping over time (Figure 4D,E). Interestingly, there was also less titer variation observed at the longest incubation period than at the two shorter periods. Infection prevalence was also significantly reduced at 15 days compared to 5 or 10 days (Figure 4F). These results suggest that incubation period, but not virus concentration, can affect the amount of variation in individual mosquito virus titer, with reduced titer variation observed at 15 days PBM.

### 3.6. No Correlation between Virus Titer and Either the Weight of Mosquito or the Amount of Blood Ingested

**Figure 5:**
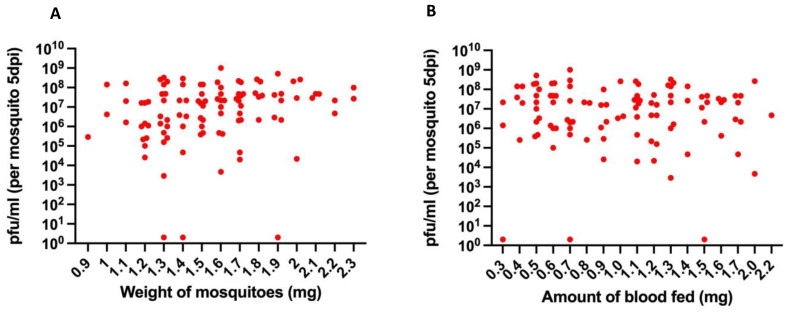
Correlation analysis to determine the correlation between mosquito weight or amount of blood fed and virus titer at 5 d PBM. (**A**) Correlation analysis of mosquito weight and virus titer. (**B**) Correlation analysis of amount of blood ingested and virus titer. Sample size used was 100. Pearson’s correlation chi square test was used for the statistical analysis.

Since mosquito size has been shown to affect the arbovirus titer [14], we considered the possibility that size variation between individual mosquitoes within a single experiment could be an underlying cause of variation in titer between individual mosquitoes. To test this, we measured mosquito size by weighing Orlando mosquitoes immediately after they had fed on blood containing 1.1 × 10^9^ PFU/mL of MRE-16 and then weighing the same mosquitoes again 3 days later, after the blood meal was digested. In a separate experiment involving 13 mosquitoes, comparing mosquito weight before blood feeding and at 3 days PBM indicated there was a small but mostly consistent weight gain during this time period, ranging from 0 mg to 0.3 mg with an average of 0.15 mg (Table S1). Thus, for the purposes of this comparison, the weight after 3 days was assumed to be representative of the mosquito weight at the time of feeding. Results from this experiment showed that mosquito weight did not correlate with the virus titer (*p*-value = 0.961) (Figure 5A).

Another possible source of individual variation could be the amount of blood (and thus virus) ingested. In all experiments, we routinely selected only mosquitoes that appear to have taken a full blood meal, as assessed by the amount of blood visible in the abdomen. However, to determine whether the amount of blood ingested contributes to variation in titer, the amount of blood ingested by the mosquitoes in Figure 5A was determined by the difference in weight immediately after blood feeding versus 3 days later. The amount of ingested blood ranged from 0.3 mg to 2.2 mg. However, there was not a significant correlation between the amount of blood ingested and the virus titer (*p*-value = 0.922) (Figure 5B). Together, these results indicate that the variation in virus titer that we routinely observe after oral infection is not due to either variation in mosquito size or the amount of ingested blood.

### 3.7. Virus from High Titer Mosquitoes Replicates to a Higher Titer in Cell Culture Than Virus from Low Titer Mosquitoes

**Figure 6:**
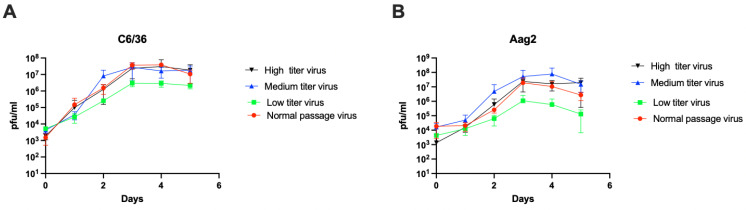
Growth curves of viruses obtained from high, medium, and low titer mosquitoes compared to normally passaged MRE-16 stock virus in (**A**) C6/36 cells and (**B**) Aag2 cells as determined by TCID_50_ assay. Viruses from pools of 5 mosquitoes were used in this experiment. The bars indicate the means ± standard error of four independent biological replicates (four pools of mosquitoes per treatment). Results were analyzed by two-way ANOVA for C6/36 (*p* = 0.0058) and Aag2 (*p* = 0.0653).

Although the virus stocks used in this study were obtained from infectious cDNA clones and passaged only twice before use, any time a virus sample is prepared, it will contain a swarm of viruses that differ from each other in their genome sequence. In addition, severe genetic bottlenecks occur during both midgut infection and midgut escape, raising the possibility that variation in titer could be due to founder effects caused by mosquitoes becoming infected with different sequence variants [27]. To begin to address this possibility, we collected the virus from pools of five homogenized mosquitoes that had low titer (2.1 × 10^4^), medium titer (2.9 × 10^6^), or high titer (2.0 × 10^8^) infections in order to determine if these virus populations differed in replication kinetics. We first used these virus populations to infect C6/36 and Aag2 cells, with a P2 stock of MRE-16 prepared by our normal procedure serving as a control. To eliminate the possibility that potential inhibitors present in mosquito homogenate could affect virus replication, medium and high titer virus stocks were serially diluted with mosquito hemogenate (rather than DMEM alone) to achieve the desired MOI. The results from the growth curves show that viruses collected from low titer mosquitoes replicated to a lower titer when compared to viruses from high titer or medium titer mosquitoes or stock MRE-16 virus (Figure 6A,B).

To further test whether mosquito homogenate could affect virus growth kinetics, high titer virus stocks were diluted with DMEM alone to reach the desired MOI. This change in procedure did not affect the replication of virus from high titer mosquitoes (compare Figure S3 to Figure 6).

### 3.8. The Virus Obtained from High Titer Mosquitoes Reinfects Mosquitoes Less Efficiently Than Normally Prepared Virus Stocks

**Figure 7:**
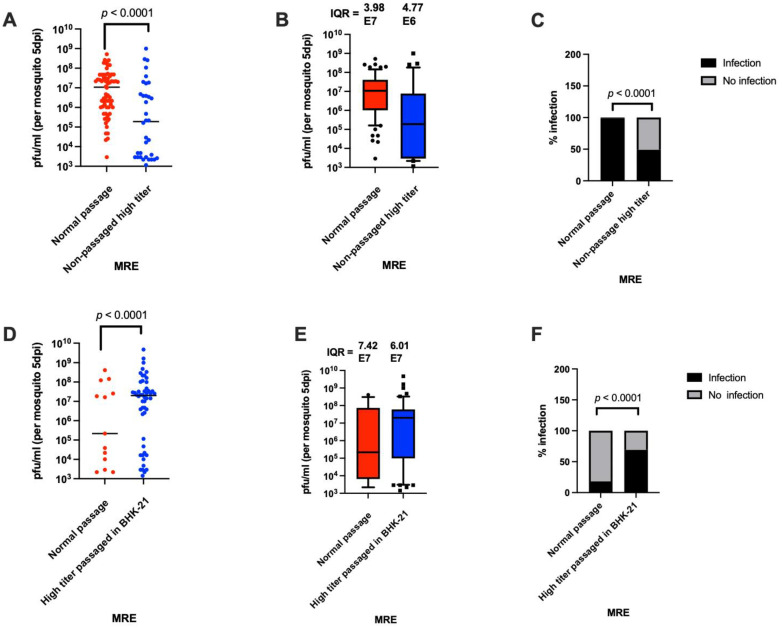
Refeeding experiment using viruses obtained from a single high titer mosquito, either passaged once in BHK-21 cells or not passaged. (**A**) Titers of mosquitoes at 5 days PBM after oral infection with virus (2.0 × 10^9^ PFU/mL) from high titer mosquitoes compared to stock MRE-16 virus. (**B**) The use of IQR as a measure of variation. The data used in panel B are the same as in A. Sample size was 72 per treatment. (**C**) Prevalence of infection after oral infection with virus from high titer mosquitoes versus stock MRE-16 virus. (**D**) Titers of mosquitoes at 5 days PBM that were orally infected using virus from high titer mosquitoes passaged once in BHK-21 cells versus MRE-16 stock virus (1 × 10^6^ PFU/mL). (**E**) The use of IQR as a measure of variation. The data used in panel (**E**) are the same as in (**D**). (**F**) Prevalence of oral infection after exposure to virus from high titer mosquitoes passaged once in BHK-21 cells versus stock MRE-16 virus. Sample size used was 72 per treatment. Statistical analysis was performed as described for Figure 3.

To further examine possible differences in replication, viruses isolated from high titer mosquitoes were used to reinfect mosquitoes either after being passaged once in BHK-21 cells or without passage. If the high virus titer was due to founder effects in the first experiment, we would expect to see a skewing of virus titers towards higher values when the virus from high titer mosquitoes was used in a subsequent oral infection. The viral concentration for both high titer mosquitoes used for the re-infection experiment without passage in BHK-21 cells was 1.0 × 10^9^ PFU/mL. The same concentration of normal stock virus was used to orally infect mosquitoes. The results from this experiment show that the non-passaged virus from high titer mosquitoes replicated to significantly lower median titer than the stock virus (Figure 7A,B). The prevalence of infection was also significantly lower for the non-passaged high titer mosquitoes than for the stock virus (99% versus 49%) (Figure 7C).

It has been previously observed that alternate host cycling (or lack thereof) between vertebrate hosts and invertebrate vectors can affect arbovirus fitness [28]. We thus passaged the virus from high titer mosquitoes once in BHK-21 cells before feeding it to mosquitoes in a blood meal. The titer of the passaged virus was 2.0 × 10^6^ PFU/mL, and so an equivalent concentration of stock virus was used as a control to orally infect mosquitoes, and the mosquitoes were titered at 5 days PBM. The results from this experiment show that the virus obtained from high titer mosquitoes and passaged in BHK-21 cells resulted in a significantly higher median titer and infection prevalence than the stock MRE-16 virus when used at this relatively low concentration (Figure 7D–F), while mosquitoes infected with either virus stock exhibited high variation in virus titer. This result further supports the hypothesis that founder effects may play a role in causing the high degree of variation in virus titer that is observed when mosquitoes are orally infected.

## 4. Discussion

To begin to explore what biological factors contribute to variation in the arbovirus titer between individual mosquitoes after oral infection, we used SINV as a model organism to study titer variation in a laboratory strain of *Ae. aegypti*. In doing so, we learned several important things about the causes of individual variation. First, the route of infection contributes to variation in the virus titer. We observed much greater variation in virus titers after oral infection than after intrathoracic infection, which is consistent with previous reports [16,17,29]. This result suggests that a major source of individual variation involves midgut infection and/or midgut escape [30]. In addition, the ability of arboviruses to successfully infect and escape the mosquito midgut is modulated by the midgut innate immune response [31]. The differential expression of the innate immune genes, such as those involved in RNA interference (RNAi) [32], Toll pathway, Jak/Stat pathway, and immune deficiency (IMD) pathway [33], can influence whether *Ae. aegypti* will be susceptible or refractory to viral infection. The gut microbiome also modulates midgut immune response by modulating the expression of antimicrobial peptides, which can influence the degree to which arboviruses successfully infect and escape the midgut [34]. Any of this myriad of factors could affect the virus titer in the body of the mosquito.

We also tested several other factors for their involvement in causing individual variation. Importantly, we showed that the virus titer in *Ae. aegypti* did not correlate with either the size of the mosquito or the amount of blood ingested. Reports from [14] show that although large *Ae. aegypti* mosquitoes were significantly more susceptible to Ross River virus than small mosquitoes when fed with viremias of varying titers, the difference in susceptibility was less apparent at higher viremias. Since high virus concentration was used in this study, it may partially explain why there was no correlation between size of mosquito and viral titer. This result suggests that some other intrinsic factor is responsible for individual variation in virus titer, such as the strength of the midgut immune response [33].

We further considered whether the degree of individual titer variation that is observed is affected by other factors. Our results indicate that the amount of individual variation is not affected by mosquito age or by the concentration of the virus in the blood meal. Furthermore, high levels of individual titer variation were observed when either one of two different strains of SINV, MRE-16 and TE, was used to infect *Ae. aegypti*, and when MRE-16 was used to infect two different *Ae. aegypti* lines, Orlando and Rex-D. A sharp contrast between the median titer and prevalence of infection was observed for MRE-16 versus TE, which was expected since TE is better adapted to replicate in culture [24] than to infect mosquitoes [22,23], but both viruses resulted in high titer variability. In contrast, the incubation period did affect the amount of individual variation in virus titer, with a longer incubation resulting in decreased individual variation as well as decreased median titer and infection prevalence. This result suggests that the mosquito immune system, if given enough time, can bring down the titer of the virus [35].

To begin to determine whether there were any differences between the virus populations that accumulated during infection of high, medium, and low titer mosquitoes, we examined the replication dynamics of these virus populations in mosquito cell lines. Results from this experiment showed that the virus obtained from low titer mosquitoes replicated slower and to a lower titer than the virus from high titer or low titer mosquitoes or standard P2 virus, suggesting that there may be genetic differences between these virus populations.

To further examine this possibility, refeeding experiments were performed using virus populations obtained from high titer mosquitoes that were either amplified in BHK-21 cells or not. Attempts to perform a refeeding experiment using low titer virus proved futile because we were not able to reinfect mosquitoes or rescue the virus from low titer mosquitoes in culture, presumably due to its low titer. However, results from feeding non-passaged high titer virus to mosquitoes resulted in similar titer variability compared to the P2 stock virus but had a lower median titer and lower prevalence of infection. In contrast, results from the refeeding experiment using the high titer virus that was passaged once in BHK-21 cells showed similar variability compared to the P2 virus but resulted in a higher median titer and higher prevalence of infection than the stock P2 virus. The lower median titer and lower prevalence of infection of the P2 virus compared to other experiments in this study was likely due to the low virus concentration used in this experiment. It was interesting, however, that the virus derived from high titer mosquitoes that had been passaged once was better able to infect mosquitoes and resulted in a higher median titer than the stock P2 MRE-16. This may be attributable to genetic changes in the genome of the virus derived from high titer mosquitoes, which calls for future investigation to ascertain if this is the case. The results from these experiments also show that the virus that came from high titer mosquitoes still caused highly variable virus titers when fed to additional mosquitoes, suggesting that there still is genetic variation in this virus population, and genetic bottlenecks could still result in founder effects [27]. The ability of the virus derived from high titer mosquitoes to reinfect mosquitoes better after they are passaged in BHK-21 cells is consistent with previous reports that arboviruses need to go through the arthropod–mammalian–arthropod transmission cycle for effective infection [36,37]. This result is also consistent with literature indicating that arboviruses alternate between arthropod and vertebrate hosts to maintain their genetic stability to be able to replicate effectively in nature [37].

In summary, results from this study show that a high degree of individual titer variation is observed when even a highly inbred laboratory strain of *Ae. aegypti* is orally infected with SINV. Of the several biological factors tested, only the incubation period affected this variation in the SINV titer. The reduced titer variation that was observed due to a longer incubation period may be due to the adaptation of SINV in *Ae. aegypti*, the action of the mosquito immune system in limiting virus replication, or a combination of both processes. Variation in individual virus titer was significantly affected by the route of infection, leading us to conclude that the midgut appears to be an important tissue contributing to the variation in titer. Our findings also suggest that founder effects, due to the action of bottlenecks on the collection of virus variants present in a SINV population, may contribute to the variability in titer between individually infected mosquitoes. Deep sequencing of the virus derived from high and low titer mosquitoes may help resolve this question. However, consistent genetic changes may be difficult to detect between virus populations obtained from high and low titer mosquitoes since mutations continue to occur in the viral genome as the virus replicates after escaping from the midgut. It also remains possible that variation in the strength of the immune response between individual mosquitoes could play a role in the highly variable titer that is observed after oral infection. We are currently carrying out additional experiments to distinguish these possibilities.

## Data Availability

The data are contained within the published article and supplementary materials.

## Supplementary Materials

The following supporting information can be downloaded at: https://www.mdpi.com/article/10.3390/v14010131/s1, Figure S1: Oral infection of different batches of mosquitoes, Figure S2: Oral infection of mosquito emerging on different days, Figure S3: Growth curves of viruses obtained from low and high titer mosquitoes, Table S1: Weight of mosquitoes before blood feeding and at three days post-blood meal.
