# Supplementary material for: Investigation of Biological Factors Contributing to Individual Variation in Viral Titer after Oral Infection of Aedes aegypti Mosquitoes by Sindbis Virus
Source: Viruses. 2022 Jan 12;14(1):131. doi: 10.3390/v14010131 (PMC8780610; doi:10.3390/v14010131)
Supplement: Supplementary file 1 [file viruses-14-00131-s001.zip › viruses-1537640-supplementary.pdf]

viruses

Supplemental materials

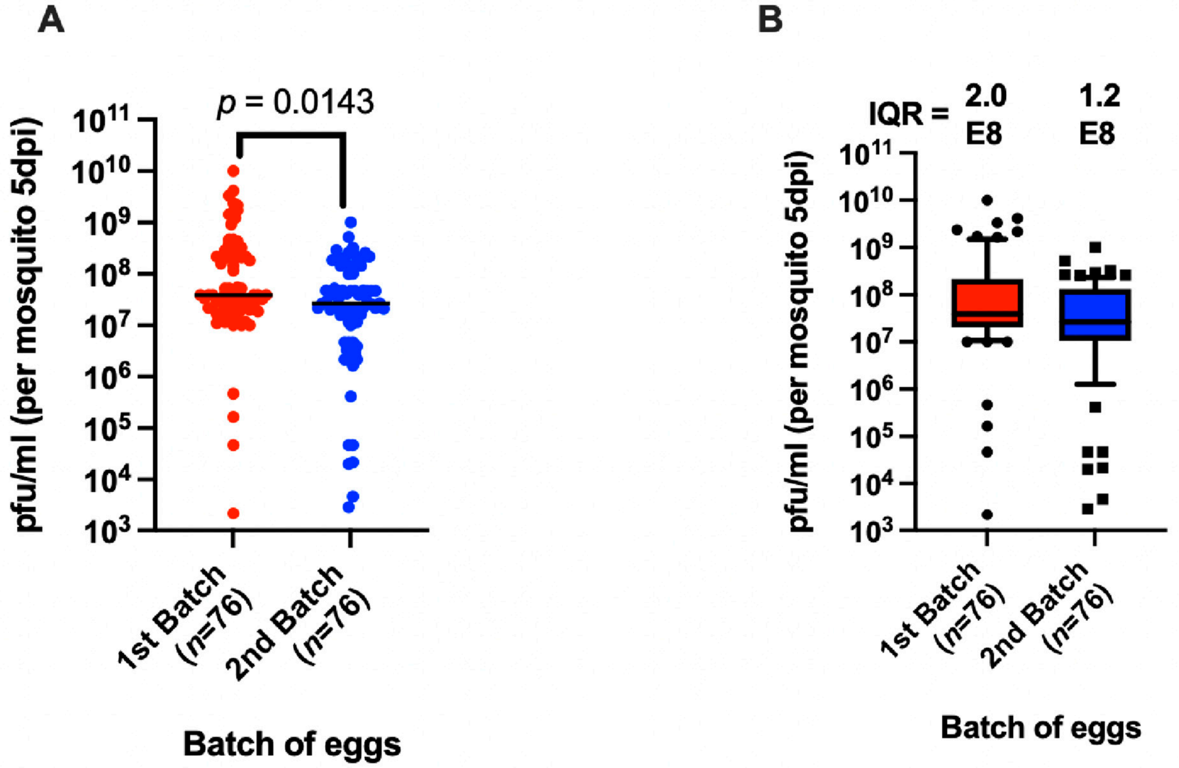

Figure S1. Oral infection of different batches of mosquitoes. (**A**) Titers of mosquitoes produced from two different batches of eggs after oral infection. (**B**) The use of IQR as a measure of variation in different batches of mosquitoes after oral infection. The dataset used in (**B**) is the same as (**A**). Statistical analysis was as described in Figure 1.

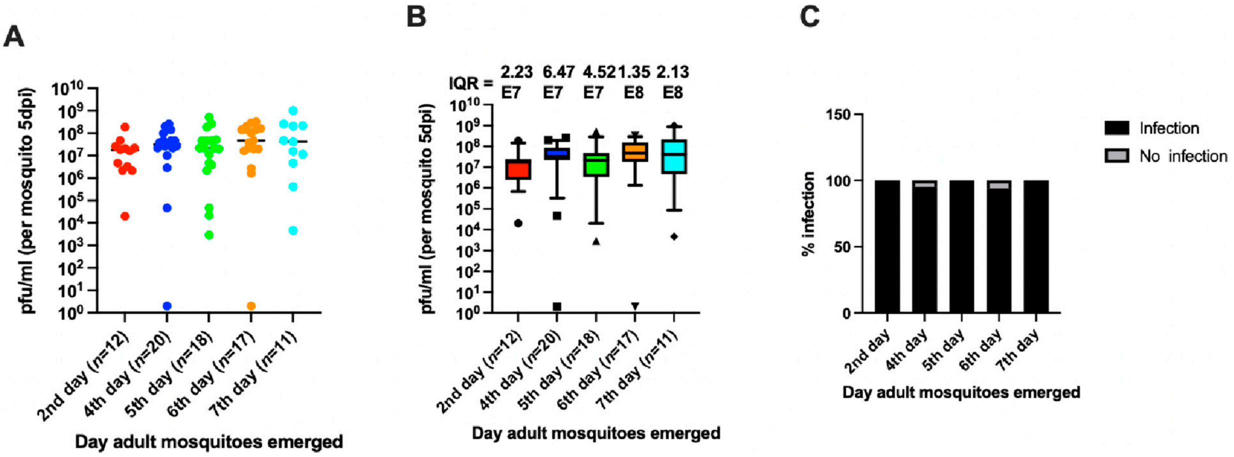

Figure S2. Oral infection of mosquitoes emerging on different days. (**A**) Titers of orally infected mosquitoes that emerged on different days, measured at 5 days PBM. (**B**) The use of IQR as a measure of variation, using the same data set as (**A**). (**C**) Prevalence of infection of adult mosquitoes emerging on different days. Statistical analysis used was as described in Figure 2.

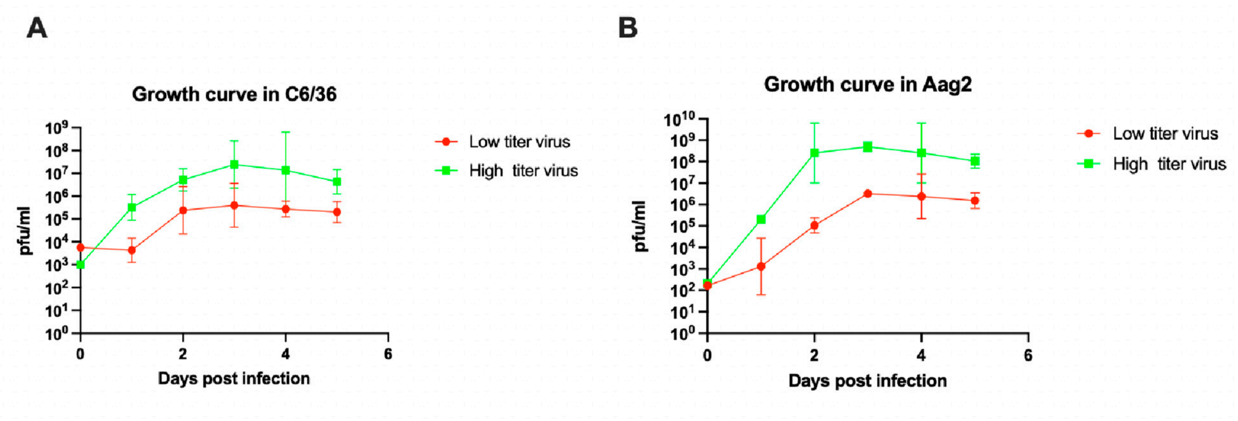

Figure S3. Growth curves of viruses obtained from low and high titer mosquitoes and diluted using DMEM in (**A**) C6/36 cells and (**B**) Aag2 cells as determined by $TCID_{50}$ assay. The bars indicate the means ± standard error of two independent experiments.

Table S1. Weight of mosquitoes before blood feeding and at three days post-blood meal.

Mosquito ID #	Weight Before Blood Fed (mg)	Weight 3 d PBM (mg)
U1	1.5	1.7
U2	1.8	2.1
U3	1.5	1.5
U4	1.6	1.8
U5	1.7	1.8
U6	1.5	1.6
U7	1.3	1.6
U8	1.9	2.0
U9	2.1	2.3
U10	1.3	1.4
U11	1.5	1.7
U12	1.6	1.7
U13	1.4	1.5
